# Characteristics of S100B and Neuron Specific Enolase in Differentiating Acute Vertigo Cases with Central Cause; a Diagnostic Accuracy Study

**Published:** 2020-01-28

**Authors:** Javad Mozafari, Hassan Motamed, Kambiz Masoumi, Mohammad Ghasem Hanafi, Mohammad Ali Fahimi, Zahra Derakhshani, Farzaneh Ehyaie

**Affiliations:** 1 Department of Emergency Medicine, Golestan General Hospital, Ahvaz Jundishapur University of Medical Sciences, Ahvaz, Iran.; 2 Department of Emergency Medicine, Imam Khomeini General Hospital, Ahvaz Jundishapur University of Medical Sciences, Ahvaz, Iran.; 3 Department of Radiology, Ahvaz Jundishapur University of Medical Sciences, Ahvaz, Iran.; 4 Student Research Committee, Ahvaz Jundishapur University of Medical Sciences, Ahvaz, Iran.

**Keywords:** Vestibular neuronitis, biomarkers, S100B protein, human, phosphopyruvate hydratase, vertigo

## Abstract

**Introduction:**

Differentiating central vertigo from peripheral ones poses a challenge to specialists. The present study aimed to examine the potential screening value of S100B and neuron-specific enolase (NSE) in this regard.

**Methods:**

This prospective cross-sectional study recruited adult acute vertigo patients with suspected central causes visiting the emergency department (ED) in the first six hours since the onset of symptoms. The screening performance characteristics of S100B and NSE biomarkers in differentiating central vertigo cases were measured considering brain magnetic resonance imaging (MRI) as the reference test.

**Results:**

85 cases who met the criteria were enrolled to the study (82.3% female). The MRI of 21 (24.7%) cases had abnormal findings. The two groups were the same in terms of age, sex, and vital signs. Patients with abnormal brain MRI had significantly higher levels of S100B (p < 0.001) and NSE (p < 0.001). S100B and NSE had area under the receiver operating characteristic (ROC) curve of 90.3 (95% CI: 80.7 – 99.8) and 96.9 (95% CI: 93.7 – 100.0) in differentiating the central causes of acute vertigo, respectively. At the cut-off point of above 119.68 pg/l, S100b had sensitivity of 90.00% (95% CI: 78.83 –95.86) and specificity of 92.00% (95% CI: 72.49 – 98.60). The sensitivity and specificity of NSE at the cut-off point of above 18.12 ng/ml were 100.00% (95% CI: 93.14 – 100.00) and 89.47% (95% CI: 65.46 – 98.15), respectively.

**Conclusion:**

The serum levels of S100B and NSE were significantly higher in patients with central vertigo, and could therefore be considered as accurate tools in screening acute vertigo cases with central causes in ED.

## Introduction:

Vertigo is a common cause of visits to the emergency department (ED), with a prevalence of 40% in those aged above 40 years (1). Given its numerous causes, differentiating central vertigo from peripheral ones poses a challenge to specialists (2). 

Central causes and brain vascular accidents associated with vertigo are life-threatening and it is vital to find a quick and accessible method for diagnosing central vertigo and posterior cerebral circulation stroke in the ED (3). Since the vertebra-basilar circulation supplies important structures such as the brain stem (4), cerebellum, and ventricular and inner ear cochlear structures, acute isolated vertigo may be caused by lack of circulation or stroke in the midbrain (5). A rapid and timely diagnosis of brain ischemia as an emergency cause of vertigo can accelerate therapeutic measures and improve prognosis (6). So far, brain magnetic resonance imaging (MRI) has been the best diagnostic method for diagnosing the cause of vague vertigo (7). Yet, MRI cannot be used for all patients with unclear diagnosis as it is not always accessible and is costly (8). 

Neuron-specific enolase (NSE) is a candidate biomarker for central nervous system (CNS) damage that passes the blood-brain barrier (BBB) (9). Its neuropath logical levels occur in head trauma accidents or neurological diseases. The normal level of NSE can reliably rule out major CNS pathologies (10).

The main advantage of using NSE is that its increased serum or cerebrospinal fluid (CSF) concentration can be a sensitive tool for determining CNS damage at a molecular level before gross changes emerge. An increase in NSE level has been reported before any detectable changes in intracranial pressure, neuroimaging, and neurological examination findings (11, 12).

Another biomarker of CNS damage proposed in the last decade as a peripheral marker of BBB permeability is the calcium-binding protein S100B (13, 14). Few studies have measured it in patients complaining of acute vertigo presenting to ED in order to differentiate peripheral vertigo from that caused by posterior circulation stroke (15).

Serum biomarkers may be effective in determining the need for imaging. The present study aimed to examine the potential screening values of S100B and NSE in differentiating true vertigo cases with central causes in the ED.

## Methods:

***Study Design and Setting***

This prospective cross-sectional study recruited adult acute vertigo patients with suspected central causes visiting the ED of Golestan Hospital, Ahvaz, Iran from 2017 to 2018, within the first six hours since the onset of symptoms. The declaration of Helsinki for research involving human subjects was considered and the Ethics Committee of Ahvaz Jundishapur University of Medical Sciences (IR.AJUMS.REC.1395.529 and IR.AJUMS.REC.1396.1033) approved the study protocol. All patients provided written informed consent before entering the study.

***Participants***

Patients above 18 years old, with chief complaint of acute vertigo, negative history of vertigo or idiopathic cranial or auditory system pathologies that are classified as central vertigo, and patients without any persistent neurological deficits like weakness or unsteadiness were included. Patients not willing to participate were excluded. History of head trauma, disorders on the electrocardiogram (ECG) such as conductive disorders, dysrhythmia, or cardiac ischemia, other neurological exams besides vertigo during examination, visiting later than six hours after the onset of vertigo, having intracranial pathologies in previous radiological examination (e.g. space-occupying masses or previous surgery), contraindication for performing brain MRI, diseases such as pulmonary Squamous-cell carcinoma (SCC), neuroblastoma, melanoma, seminoma, Merkel cell carcinoma, tumors, carcinoids, teratoma, malignant pheochromocytoma, Guillain–Barré syndrome, and Creutzfeldt-Jakob disease were also among the exclusion criteria.

***Study Protocol***

Data were gathered from all eligible patients, initial examinations were performed, and ECGs were obtained. Soon after initial stabilization, in addition to obtaining regular venous blood samples, a venous blood sample was taken by the ED nurse (trained research assistant) specifically for S100B and NSE biomarkers, and the time of sampling was recorded. Then, patients were referred to the imaging department for brain MRI. 

Blood samples (10 ml) were taken in gel tubes and rested for 30 minutes to clot. The samples were then centrifuged for 10 minutes at 800 to 1000 rpm in the ED laboratory. Serum samples were diluted with 1 ml of distilled water and then transferred to test tubes. The preliminary sample was sent to the laboratory for measuring the biomarkers. Prior to the final analysis, the samples were maintained at -20 °C; they were assayed separately using electrochemiluminescence method. Furthermore, all laboratory personnel were blinded to patient data and imaging findings of the two groups.

Preliminary brain diffusion-weighted MRI (DWI) of all patients was separately ordered by an emergency medicine specialist and performed using a single MRI machine and interpreted by a neurologist and a radiologist, both blinded to the biomarker results. Serum samples were taken within a maximum of six hours after the onset of vertigo. Finally, patients were divided into two groups of positive and negative MRI findings, and the levels of biomarkers were compared across the two groups. 

***Statistical Analysis***

All data were analyzed using SPSS, described using mean and standard deviation (SD) for quantitative, and frequency and percentage for qualitative variables. T-test or Mann Whitney U test as well as Chi-squared test were used for analyzing the data. Area under the receiver operating characteristic (ROC) curve was calculated in order to determine the predictive value of S100B and NSE and the optimal cut-off point of S100B and NSE for distinguishing central vertigo based on the best sensitivity and specificity. All results are reported with 95% confidence interval (CI) and p-value of <0.05 was considered significant.

## Results:

***Baseline characteristics of studied patients***

**Figure 1:**
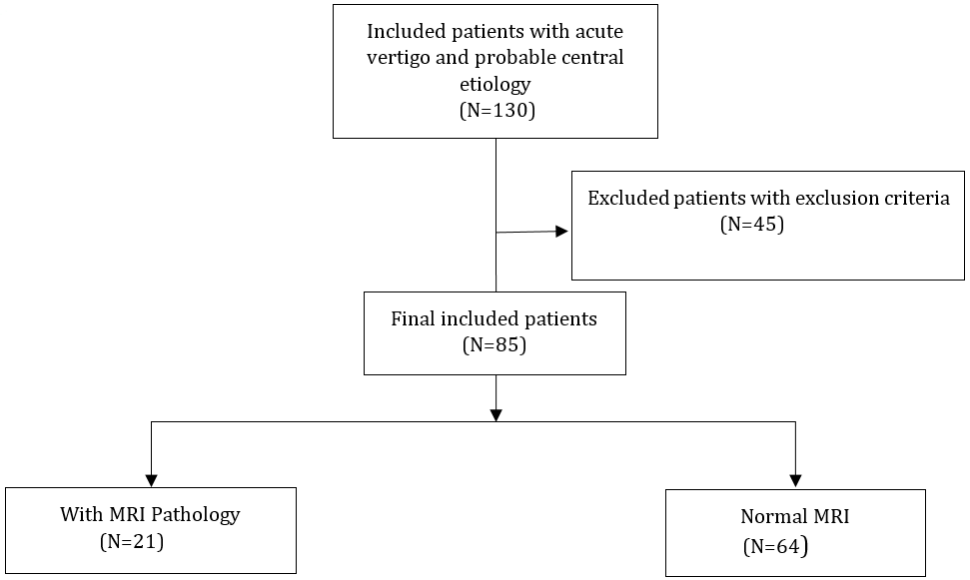
STARD flowchart diagram

130 patients with acute vertigo and possible central causes were evaluated and finally 85 cases who met the criteria were enrolled in the study (82.3% female; Figure 1). The MRI of 64 (75.3%) cases was normal and the other 21 patients had chronic ischemic changes. Mean age of the patients was 53.06±16.45 years in the normal MRI group and 49.63±14.74 years in the abnormal group. Baseline characteristics of patients in both groups are given in Table 1. The two groups did not differ in terms of age, sex, and vital signs. Patients with abnormal brain MRI findings had significantly higher levels of S100B (p < 0.001) and NSE (p < 0.001).

**Table 1:** Comparing the baseline characteristics of patients with positive and negative brain magnetic resonance imaging (MRI) findings

**Variables**	**Brain MRI Findings**		P value
	**Positive (n=21)**	**Negative(n=64)**	
**Gender (female)**	10 (47.6)	60 (93.7)	0.073
**Age (years)**	49.63 ± 14.74	53.06 ± 16.45	0.398
**Vital signs**			
SBP (mmHg)	141.12 ± 17.03	138.48 ±21.91	0.616
DBP (mmHg)	84.09 ± 10.98	77.07 ±12.17	0.021
RR (/minute)	17.05 ± 2.04	16.80 ±1.34	0.383
PR (/minute)	79.00 ± 5.70	80.53 ±5.88	0.300
Temperature (C)	36.50 ± 0.321	36.46 ±0.32	0.603
SpO_2_ (%)	98.08 ±0.97	97.91 ±0.93	0.484
**Biomarker level**			
S100B (pg/ml)	217.13 ± 119.28	77.39 ±31.67	<0.001
NSE (ng/ml)	30.90 ± 7.34	10.92 ±6.34	<0.001

Data are presented as mean ± standard deviation or number (%). SBP: systolic blood pressure; DBP: diastolic blood pressure; RR: respiratory rate; PR: pulse rate; SpO_2_: peripheral capillary oxygen saturation; NSE: neuron specific enolase.

**Figure 2:**
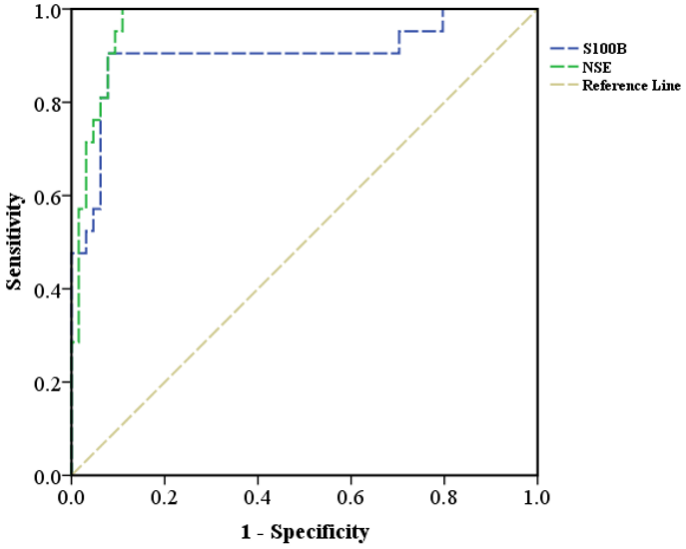
The area under the receiver operating characteristic (ROC) curve of S100B and neuron specific enolase (NSE) biomarkers in differentiating acute vertigo cases with central cause

**Table 2:** Screening performance characteristics of S100B and neuron specific enolase (NSE) biomarkers in differentiating acute vertigo cases with central cause

**Characteristics**	**S100B**	**NSE**
True positive	54	66
True negative	23	17
False positive	2	2
False negative	6	0
Sensitivity	90.00 (78.83 - 95.86)	100.00 (93.14 – 100.00)
Specificity	92.00 (72.49 - 98.60)	89.47 (65.46 - 98.15)
Positive predictive value	96.42 (86.61 – 99.37)	97.05 (88.83 – 99.48)
Negative predictive value	79.31 (59.73 – 91.29)	100.00 (77.07 – 100.00)
Positive likelihood ratio	27.00 (6.91 -105.39)	33.00 (8.41 – 129.34)
Negative likelihood ratio	0.26 (0.12 – 0.54)	0.00 (0.00 – NaN)
Total accuracy	90.3 (80.7- 99.8)	96.9 (93.7 - 100.0)

Data are presented with 95% confidence interval (CI). NaN: the calculation cannot be performed because the values entered include one or more instances of zero.

***Screening value of studied biomarkers***

Table 2 summarizes the screening performance characteristics of S100B and NSE biomarkers in differentiating the central causes of acute vertigo. S100B had the area under the ROC curve of 90.3 (95% CI: 80.7 – 99.8) for differentiating the causes of acute vertigo (figure 2), and at the cut-off point of above 119.68 pg/l, had sensitivity of 90.00% and specificity of 92.00%, in detecting cases with abnormal MRI findings (central cause of vertigo). In addition, the NSE biomarker in patients with acute vertigo had the area under the ROC curve of 96.9 (95% CI: 93.7 – 100.0; figure 2), and at a cut-off point of above 18.12 ng/ml, had sensitivity of 100.00% and specificity of 89.47%, indicating the central nature of vertigo. 

## Discussion:

Our findings revealed that S100B and NSE have acceptable screening performance characteristics in differentiating acute vertigo cases with central causes. 

The cause of vertigo may be central (brainstem, cerebellum, or brain involvement) or peripheral (vestibule-cochlear nerve or inner ear labyrinth), systemic (cardiac or metabolic diseases) or psychological (e.g. anxiety). Central vertigo may be dangerous and even lead to mortality. Yet, it is often difficult to diagnose the cause of vertigo, and only MRI imaging has so far been used as the gold standard for diagnosing these cases (16).

Kartal et al. examined the serum level of S100B in 82 acute vertigo patients within six hours since the emergence of symptoms and reported a sensitivity of 83.9% and specificity of 51% for diagnosing the central cause of vertigo in cases of serum concentration of above 30 pg/ml (15). 

Our results showed that serum level of S100B and NSE biomarkers were significantly higher in patients with abnormal MRI findings. Considering the high sensitivity and specificity at the cut-off point of 19.2 ng/ml, it seems that NSE is more valuable than S100B in differentiating peripheral and central causes of vertigo. 

In the study by Bharosay et al., NSE level was less than 25 ng/ml in the control group (n=101) and above 25 ng/ml in patients with ischemic stroke (n=70)(17). 

Moreover, in a study by Bandhyopadhyay et al. on 79 patients with head trauma and GCS<13, blood samples were taken in the first 3.8 hours on average; seven patients with poor outcome and GCS of <5 had the NSE level of 46.4±12.7 ng/ml, and patients with good outcome had the NSE level of 19.5±1.4 ng/ml. In cases of serum concentration of above 21.2 ng/ml, NSE had the sensitivity of 86% and specificity of 74% in predicting poor outcome (18). 

Fridriksson et al. studied patients with head trauma, and reported that the serum level of NSE was higher in 22 patients with abnormal head computed tomography (CT) scan (26.7±21.4 ng/ml) compared to 28 patients with normal CT (17.7±7.8 ng/ml); serum concentration above 15.3 ng/ml had sensitivity of 77% and specificity of 52% in predicting the presence of abnormal brain CT finding (18). 

Although patients with head trauma and reduced level of consciousness were excluded in our study, positive MRI findings may be indicative of neurological damage, probably consistent with the probability of abnormal CT in the reviewed studies. 

Measuring this biomarker is not costly, it can quickly help diagnose patients suspected of central vertigo in ED, it can reduce hospital stay and costs, and it is easily accessible everywhere compared to MRI. Therefore, it can be used as a screening test with an acceptable accuracy in cases where differentiating central from peripheral causes is difficult. Those with positive test results using this method can be the final candidates for MRI. Of course, studies on larger samples are required in order to determine a more precise cut-off point. 

## Limitations

The number of patients with normal MRI findings did not match the number of patients with abnormal MRI findings. A relatively small sample size was another limitation.

## Conclusion:

The serum levels of S100B and NSE were significantly higher in patients with central vertigo, and could therefore be considered as accurate tools in screening acute vertigo cases with central causes in ED. It is clear that high-risk cases should be confirmed with brain MRI as the gold standard tool in this regard.
